# Sustained efficacy of artesunate-sulfadoxine-pyrimethamine against *Plasmodium falciparum* in Yemen and a renewed call for an adjunct single dose primaquine to clear gametocytes

**DOI:** 10.1186/s12936-016-1344-0

**Published:** 2016-05-27

**Authors:** Wahib M. Atroosh, Hesham M. Al-Mekhlafi, Georges Snounou, Adel Al-Jasari, Hany Sady, Nabil A. Nasr, Yee-Ling Lau, Johari Surin

**Affiliations:** Department of Parasitology, Faculty of Medicine, University of Malaya, 50603 Kuala Lumpur, Malaysia; Department of Microbiology and Parasitology, Faculty of Medicine and Health Sciences, University of Aden, Aden, Yemen; Endemic and Tropical Diseases Unit, Medical Research Center, Jazan University, Jazan, Kingdom of Saudi Arabia; Department of Parasitology, Faculty of Medicine and Health Sciences, Sana’a University, Sana’a, Yemen; UPMC Université Paris 06, Inserm (Institut National de la Santé et de la Recherche Medicale), Centre d’Immunologie et des Maladies Infectieuses (Cimi-Paris), UMR 1135, ERL CNRS 8255 (Centre National de la Recherche Scientifique), Sorbonne Universités, 91 Boulevard de l’Hôpital, F-75013 Paris, France; National Malaria Control Programme, Ministry of Public Health and Population, Sana’a, Yemen; Centre for Research and Innovation, Taylor’s University, 47500 Subang Jaya, Selangor Malaysia

**Keywords:** Artemisinin-based combination therapy, Sulfadoxine-pyrimethamine, Drug resistance, Malaria, *Plasmodium falciparum*, Gametocytaemia, Kelch 13-propeller, Yemen

## Abstract

**Background:**

In Yemen, artesunate plus sulfadoxine-pyrimethamine (AS + SP) has been used as first-line treatment for uncomplicated falciparum malaria, which accounts for about 99 % of malaria cases. There is evidence that resistance to SP is increasing, with potential negative impact on efficacy, and in particular on curbing transmission. This study aims: (a) to evaluate the therapeutic efficacy of AS + SP treatment for uncomplicated falciparum malaria in Yemen; (b) to investigate the frequency of mutations in *Plasmodium falciparum* genes associated with resistance to AS (Kelch 13 propeller domain, *pfK13*) and SP (dihydrofolate reductase, *pfdhfr*, and dihydropteroate synthase, *pfdhps*); and (c) to assess the adequacy of this ACT to clear gametocytes.

**Methods:**

A 28-day in vivo evaluation of the clinical and parasitological response to three-day course of AS + SP was carried out in two areas of high endemicity (Hodeidah and Al-Mahwit provinces, Tehama region) in Yemen according to standard WHO protocol 2009. Clinical and parasitological indices were monitored over a 28-day follow-up, and the outcome was PCR-corrected. The frequencies of mutations in the *pfdhfr*, *pfdhps*, and *pfK13* genes were obtained by sequencing following amplification.

**Results:**

Eighty-six patients completed the study, with a cure rate of 96.5 % (94.2 % PCR-uncorrected). Whereas four (4.7 %) patients still showed parasitaemia on day 2 post-treatment, all were found negative for asexual malaria stages on days 3 and 7. The efficacy of gametocyte clearance was poor (14.5, 42.5 and 86.0 % on days 7, 14 and 28, respectively), with gametocytes persisting throughout the study in some patients. All the isolates sequenced had the *pfk13* propeller domain wild-type allele, and mutations associated with SP failure were observed only for *pfdhfr* with the double mutation (S108N + N51I) found in 65.4 % of the isolates sequenced.

**Conclusion:**

In Yemen, AS + SP therapy remains effective for the treatment of uncomplicated falciparum malaria. Mutations were not detected in *pfk13* or *pfdhps*, though double mutations were observed for *pfdhfr*. The observed persistent gametocytaemia re-enforces calls to add a single dose primaquine to this ACT in order to minimizes the potential for transmission and enhance regional efforts to eliminate malaria.

**Electronic supplementary material:**

The online version of this article (doi:10.1186/s12936-016-1344-0) contains supplementary material, which is available to authorized users.

## Background

The worldwide spread of *Plasmodium falciparum* resistance to anti-malarial drugs led to the development of new strategies to treat malaria and extend the life of novel drugs. Over the last decade, artemisinin-based combination therapy (ACT) has become the cornerstone of malaria treatment policies [1, 2]. This contributed to the global decline in the number of malaria cases (18 %) and mortality (48 %) between 2000 and 2015 [2]. However, the emergence and spread in Southeast Asia of *P. falciparum* lines with reduced susceptibility to artemisinin [3–5] raises urgent concern. Mutations in the propeller domain of the parasite’s Kelch 13 protein have recently been associated with the delayed clearance that characterizes this resistant phenotype [6]. To date, at least 54 single nucleotide polymorphisms (SNPs) have been identified within this domain [5, 7–9], with C580Y and F446I the most prevalent in Southeast Asia [8, 10–12]. Numerous novel mutations in the *pfk13* propeller domain have been reported from African *P. falciparum*, but none was associated with artemisinin resistance [10, 13–16].

In Yemen, malaria is still a major life-threatening health problem for more than 60 % of the population of nearly 25 million people, and in particular in the southwestern corner of country [5]. The malaria cases are nearly all due to infection by *P. falciparum* (99 %), with *Anopheles arabiensis*, *Anopheles sargentii* and *Anopheles azaniae* as the major insect vectors [5, 17]. The countries of the Arabian Peninsula are now considered to be malaria-free [18], with two exceptions: Yemen where malaria is still prevalent with high rates of mortality and morbidity, and Saudi Arabia where the cases, principally recorded in the southwestern districts bordering Yemen are considered to be mainly imported from the bordering Tehama region of Yemen and from Sudan [19, 20]. The decline in chloroquine efficacy in Yemen [21–24] prompted the adoption in 2009 of artesunate in combination with sulfadoxine-pyrimethamine (AS + SP) as the first-line treatment of uncomplicated *P. falciparum* infections, with a combination of artemether with lumefantrine (AL) as a second-line treatment [25]. Although clinical efficacy of AS + SP treatment in Yemen has not diminished since its introduction [21, 26], there are indications from the few molecular surveys of *P. falciparum* populations in Yemen that whereas no mutations associated with drug resistance occur for *pfdhps*, the prevalence of such mutations in *pfdhfr* is increasing [27–30]. Finally, it is known that SP does not provide optimal clearance of gametocytes, even when combined with AS, prompting calls to include monitoring of the sexual stages in dug efficacy studies [31].

The present in vivo trial aimed to assess the clinical and parasitological efficacy of AS + SP treatment in an area of high endemicity close to the Saudi Arabian border, and to investigate the polymorphisms in the *pfk13* gene propeller domain, *pfdhfr* and *pfdhps* genes of the *P. falciparum* parasites circulating in this area. Furthermore, the impact of the AS + SP treatment on microscopic gametocytaemia was also evaluated throughout the follow-up period.

## Methods

### Study area and patients

**Fig. 1:**
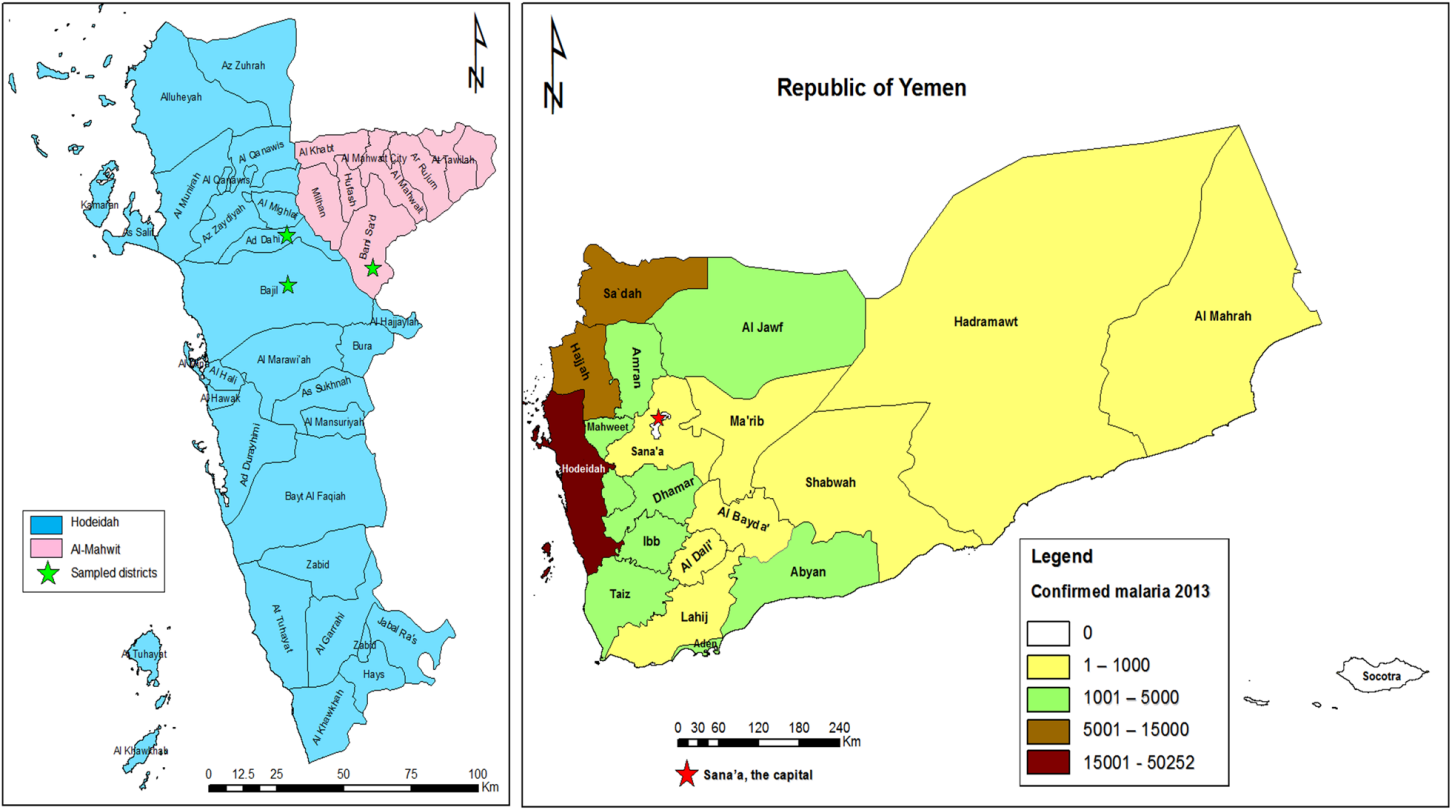
Map showing study area (Hodeidah and Al-Mahwit provinces) and the incidence of malaria in Yemen in 2013

An active case study was carried out among febrile individuals, suspected to have malaria infection, was conducted from March to May 2014 in some malaria-endemic areas in two provinces, Hodeidah and Al-Mahwit located in Tehama region, northwest Yemen (Fig. 1). The Hodeidah province (14°46′00N, 43°15′00E) is a coastal area located along the Red Sea in the western part of Yemen, about 226 km from Sana’a, the capital. It is with a total area of 117,145 sq km and a total population of about two million people [32]. The Al-Mahwit province (16.25°N, 44.717°E) is located about 111 km west of Sana’a), with a total area of 2858 sq km and a total population of about 600,000 people [32]. In Tehama region, the climate is a combination of tropical monsoon with mean temperature of 37.5 °C in summer and 24 °C in winter. The mean rainfall is 200 mm/year, occasionally during the summer while the weather is dry in winter. Malaria transmission occurs year round, with a transmission peak from January to March.

Districts and villages with high malaria endemicity were selected for this study based on the malaria incidence records for the year 2013 provided by the National Malaria Control Programme (NMCP), Yemen. These included AdDahi and Bajil Districts in Hodeidah Province, and Khamis Bani-Saad District in Al-Mahwit Province. According to the NMCP records for 2013 (Fig. 1), Hodeidah had the highest number of malaria cases (15,001–50,252 cases) followed by Sa’dah, Hajjah and Al-Mahwit provinces (northwest Yemen). However, the authors could not collect samples from these two provinces because of the state of civil war during the proposed sampling period. The research team comprised of the Principle Investigator, two medical laboratory experts and a physician visited each study area and established a station. The heads of the villages were informed about the objectives and the proposed procedures in order to obtain their permission to initiate the study. Afterwards, the village heads asked all residents to present themselves at the station where they were informed of the aims and procedures of the study, and the modalities of their participation. Those who voluntarily agreed to take part in the study were assessed for eligibility. The team also enquired about any individuals who could not attend because of illness or fever and these persons were visited at their home settings. The population in the villages sampled had minimal travel histories.

A total of 622 febrile individuals were examined in ten villages: Halalah, Al-Huamarah, Al-Meshaahra, Al-Rakib, Al-Rufae, Al-Sharjah, Deer Shareef, Kidf Zumailah, Shat Hajal, and Siraj in addition to the city of Bajil. Of these, 86 *P. falciparum*-positive patients were involved in the in vivo study.

### *Plasmodium falciparum* isolates

A finger-prick blood sample was collected from each participant for rapid diagnostic test (RDT) (CareStart™ Malaria HRP2, Cat no. G0141, Access Bio, Inc, USA), for preparing thick and thin blood smears, and to obtain blood spots on 3MM Whatman^®^ filter paper (Whatman International Ltd, Maidstone, UK). The filter papers were left to dry in air away from dust and kept in an aluminium foil pouch with desiccant silicon bags at room temperature (25–27 °C) until use. This sample obtained prior to the administration of treatment (for those that had a confirmed malaria diagnosis) was considered to be the day 0 sample.

The blood smears were Giemsa-stained prior to microscopic examination for the identification of parasite species and stages. The parasitaemia was determined using a modified procedure for counting the asexual stages. Parasites were enumerated against 300 white blood cells (WBC) with a hand tally counter and the number was then multiplied by 25 to obtain the number of parasites per µL of blood (assuming an average of 7500 WBC/µL blood). The parasitaemia was graded as low (<1000 parasites/µL blood), moderate (1000–9999 parasites/µL blood), or severe (≥10,000 parasites/µL blood). The presence of gametocytes was examined against 500 WBC; though gametocytaemia was treated as qualitative variable in data analysis. Each microscopy-positive slide and a selection of negative slides were examined by two senior specialists and the average parasite count was considered.

### In vivo test

All the patients who fulfilled the inclusion criteria, including age of 6 months and above, axillary temperature of ≥37.5 °C or history of fever within the last 72 h, microscopically confirmed as uncomplicated *P. falciparum* mono-infection, and ability to swallow oral medication were invited to participate in the in vivo trial. Details concerning the study, its benefits and any possible potential risks were fully explained. Only those who willingly agreed to participate and gave informed written and signed consents as well as declared their willingness to comply with the study protocol for the duration of the study and to comply with the study visit schedule were enrolled in the study. Patients who had signs of severe or complicated falciparum malaria or had mixed infection with another *Plasmodium* species (*P. vivax*) or had febrile conditions due to diseases other than malaria or had a history of adverse effects due to any anti-malarial drug or any other medicines were excluded from the in vivo study. Moreover, pregnant women and females with positive pregnancy test or females who were unable to or unwilling to do a pregnancy test were also excluded.

Treatment efficacy was evaluated by monitoring the clinical and parasitological parameters over a 28-day follow-up period from the day the first drug dose was administered (day 0). The methods of treatment, schedule of follow-up and analysis of outcomes were as per the WHO guidelines [33]. The drug dosage was based on body weight and, according to the national policy of malaria treatment. After treatment administration, patients were observed for at least 30 min to make sure that the medicine was not lost through vomiting or diarrhoea. If vomiting or diarrhoea occurred within 30 min of treatment, a full treatment dose was repeated. Patients then underwent regular clinical and parasitological assessment for another seven specific days after the day of first dose (day 0): days 1, 2, 3, and 7 and then weekly on days 14, 21 and 28. Patients were advised to contact the Principal Investigator at any time during the follow-up period if symptoms returned or in case of appearance of any one of these danger signs: unable to drink or breastfeed (in the case of children), severe vomiting, presenting with convulsions, lethargic or unconscious, or difficult breathing, or appearance of jaundice or black urination. Additional clinical and parasitological assessment was obtained in such cases. Patients who missed days 1 and 2 follow-up or subsequently missed one dose of the treatment were withdrawn from the study. After day 3, patients who were lost to follow-up on day 7 but were present on days 6 or 8 (likewise days 13/15, days 20/22, days 27/29), were still included in the study. However, absence from the follow-up after day 3 for more than one day led to withdrawal from the study. Treatment outcomes were classified as early treatment failure (ETF), late clinical failure (LCF), late parasitological failure (LPF), or adequate clinical and parasitological response (ACPR), according to the WHO guidelines [33].

### Treatment regimen

ACT in the form of AS + SP is the first-line treatment in Yemen for uncomplicated falciparum malaria. AS + SP used in this study was provided by WHO through NMCP: 50 mg artesunate tablets in a strip of 12 tablets (Artesunate, Batch No. AS 120901, Guilin Pharmaceutical Co Ltd, China) and sulfadoxine 500 mg/pyrimethamine 25 mg tablets in a pack of 1000 tablets (Batch No. SP IH0132, Micro Labs Ltd, The Netherlands). The drug was administered at a daily dose of artesunate 4 mg per kg of body weight (BW) for three successive days (days 0, 1 and 2) plus a single dose of 25/1.25 mg per kg BW of SP, respectively, on the first day (day 0). Additionally, NMCP provided the study with the second-line treatment AL should AS + SP therapeutic failure occur, and parenteral quinine therapy for those who might vomit the treatment twice and/or could not take the medication orally, for pregnant women during first trimester, for patients with severe or complicated malaria and in case of AL therapeutic failure.

### DNA extraction

Two to three discs (6-mm diameter) of the blood spot dried on the filter paper were cut using a flamed-sterile puncher, and were then used for DNA extraction using a Qiagen blood and tissue kit (QIAGEN, DNeasy^®^ Blood & Tissue Kit, Cat. no. 69506, Germany) according to manufacturer’s instruction. DNA was eluted using 100 µL of the elution buffer AE (10 mM Tris–Cl; 0.5 mM EDTA; pH 9.0) and kept at −20 °C until use.

### Species-specific PCR identification of malaria parasites

All malaria-positive samples from patients recruited to the in vivo study were subjected to PCR confirmation, targeting the small sub-unit ribosomal RNA genes [34] prior to any further molecular analyses. Only samples that were positive for *P. falciparum* were considered for molecular characterization of anti-malarial drug resistance. Species other than *P. falciparum* were not detected in any of the admission samples.

### *msp1, msp2* and *glurp* genotyping

Recurrent cases were distinguished from new infections (PCR-correction) according to the protocols described previously [35, 36] for the amplification and analysis of selected polymorphic domains of the merozoite surface protein-1 (*msp1*), merozoite surface protein-2 (*msp2*) and glutamate rich protein (*glurp*) genes.

Amplification of the three markers was achieved using nested PCR [37]. Amplicons were visualized in 2.5 % of agarose gel stained with Sybr^®^ safe DNA gel stain (Invitrogen, USA) using UV documenting system (Bio-Rad, Hercules, CA, USA). The allelic variants observed were then grouped into different size bins of 25 bp for *msp1* and *msp2*, and of 50 bp for *glurp*. For those cases where parasites were observed during the follow-up period, *msp1* and *msp2* allelic variants were compared between the samples obtained before and after treatment (i.e. the base line day 0 sample, and the one collected at the recurrent episode) by capillary electrophoresis in order to increase sensitivity and resolution. Forward oligonucleotide primers of secondary PCR for *msp1* (M1-KF, M1-MF and M1-RF) and for *msp2* (M2-FCF and IC-F) were labelled with 6-FAM fluorescent dye. Amplicons were analysed by the automated ABI 3730XL Genetic Analyzer and then interpreted using GeneMapper^®^ analysis software version 4.0 (Applied Biosystems, USA). Details of primers sequences and the PCR conditions used to amplify *msp1*, *msp2* and *glurp* are available in Additional file 1.

### Gene mutation of molecular markers of anti-malarial drug resistance

Selected domains of the parasite genes associated with drug resistance were amplified by conventional or nested PCR using genomic DNA purified from the *P. falciparum* isolates, and the mutations identified following direct sequencing or through restriction fragment length polymorphism (RFLP) analysis.

*pfk13* propeller domain A single run PCR using forward and reverse primers K13-F (5′-GTTGGTGGAGCTATTTTTGAAACATCTAG-3′) and K13-R (5′-GCCAAGCTGCCATTCATTTGTATC-3′) that were designed based on *P. falciparum* 3D7 kelch gene *(PF13_0238, Gene ID: 814205),* yielding 1062 bp amplicon that corresponds to nucleotides 1094–2127 (codons 364–709). The PCR products were then visualized by 1 % agarose gel stained with Sybr^®^ safe DNA gel stain under UV, then gel-purified, sequenced in both directions, and the resulting sequences aligned using BioEdit Sequence Alignment Editor Software (version 7.1.9) and compared to *P. falciparum* 3D7 Kelch 13 gene (Gene ID: 814205). All positions were evaluated for the presence of mutations. Details of the primer sequences and PCR conditions used are available in Additional file 1.

*pfdhfr* and *pfdhps.pfdhfr* was analysed for mutations at six codons (A16 V, C50R, N51I, C59R, S108N, and I164L) and *pfdhps* at seven codons (S436A/F, A437G, K540E, A581G, A613T/S, I640F, and H645P). Amplification of *pfdhfr* was achieved by a modified nested PCR protocol [38] followed by purification and sequencing of the 700 bp amplicons instead of restriction enzymes (RE) digestion. The primary reaction was performed using Amp1 (5′-TTTATATTTTCTCCTTTTTA-3′) and Amp2 (5′-CATTTTATTATTCGTTTTCT-3′) oligonucleotides pair, and the secondary amplification reaction using SP1 (5′-ATGATGGAACAAGTCTGCGAC-3′) and SP2 (5′-ACATTTTATTATTCGTTTTC-3′) [39]. The PCR protocol for *pfdhps* amplification was slightly modified: the upstream primers for both primary and secondary PCR were changed so as to yield a longer amplicon (1005 bp). PCR amplification was performed using PS1-F (5′-GAATTTTTATCCATTCCTCATG-3′) + O2 (5′-TTCCTCATGTAATTCATCTGA-3′) and PSA-F (5′-GTATACAACACACAGATATAG-3′) + O2 primers pairs for the primary and secondary amplification reactions, respectively [38]. All PCR products were visualized in 1.5 % agarose gels and the PCR products were purified, sequenced and then aligned using BioEdit Sequence Alignment Editor Software compared to DHFR (ID: 9221804) and DHPS (ID: 2655294) genes sequences of 3D7 *P. falciparum*. (Additional file 1).

### Data analysis

Data were double entered into Microsoft Office Excel 2007 spreadsheets and cross-checked for accuracy before being exported to IBM SPSS statistical package version 20 (IBM Corp, NY, USA) for data analysis. For descriptive analysis, frequency and proportion were used to present the distribution categorical variables. All quantitative variables were examined for normality by Shapiro–Wilk test before analysis. Statistical associations between point mutations and explanatory variables, including age, gender, sites, and parasitaemia, were assessed using the Chi square test or Fisher’s Exact test where applicable. Per-protocol and Kaplan–Meier survival analysis was used to evaluate the treatment outcome and to plot the decline in gametocytaemic cases after treatment among individuals who were gametocyte-positive at enrolment. Patients were censored from the per-protocol analysis of treatment outcome if they were lost to follow-up, identified with re-infection (after PCR-correction) or decided to withdraw from the study, but they were considered in the Kaplan–Meier survival analysis until the day of withdrawal. A *P* value of <0.05 was considered statistically significant.

### Ethical statement

The study protocol was approved by the Medical Ethics Committee of the University of Malaya Medical Centre, Kuala Lumpur (Ref. 974.19), and by the Ministry of Health and the NMCP in Yemen. At the villages, the residents were informed about the aims and procedures of the study. Moreover, the participants were also informed that they could withdraw from the study at any point of time without citing reasons for doing so. Afterwards, written and signed or thumb-printed informed consents were taken from adult participant or from the parents on behalf of their children before enrolment, as approved by the mentioned Ethics Committees. The consent request form was translated into Arabic language and was read entirely to the patients or their parents/guardians before they were asked to sign the document. Furthermore, permission for pregnancy testing was sought from married female participants aged 18 years and above. RDT-positive individuals were treated according to the national malaria treatment policy, Ministry of Health and Population, Yemen (as stated in Methods). Any patient who decided not to participate or to continue in the in vivo study was also treated and followed-up until total clearance of parasitaemia by day 28.

## Results

Of the 622 individuals screened, 188 (30.2 %) and 189 (30.4 %) individuals were found positive for malaria by using the CareStart™ HRP2-RDT and microscopy, respectively. However, only 89 met the inclusion criteria and consented to be enrolled in the study. All were given AS + SP and followed up for 28 days. Two patients were lost to follow-up from day 14 onwards and one patient refused to continue from day 2, leaving 86 patients (40 males and 46 females; aged between 8 months and 65 years, mean age of 12.4 years) who were successfully followed up until day 28. Admission asexual parasite levels varied from 561 to 55,555 and with mean parasitaemia of 8199 parasites/µL blood. The general characteristics of the 86 participants are shown in Table 1.

**Table 1:** General characteristics of the participants at enrolment (n = 86)

Variable	Frequency	(%)
Province		
Al-Hodeidah	28	32.6
Al-Mahwit	58	67.4
Gender		
Male	40	46.5
Female	46	53.5
Age group (years)		
<5	20	23.3
5–15	50	58.1
>15	16	18.6
History of fever	86	100.0
Weight (kg)^b^	20.0 (17.5)	–
Haemoglobin level (g/dl)^a^	10.2 (1.9)	–
Parasitaemia (asexual parasites density per μl)		
Parasitaemia on day 0^b^	5247 (9648)	–
≤999	13	15.1
1000–9999	50	58.1
≥10,000	23	26.7
Type of house		
Wooden	44	51.2
Cement	27	31.4
Cement and wood	15	17.4
Source of drinking water		
Piped water	22	25.6
Well	35	40.7
Stream/river	29	33.7
Having electricity supply	69	80.2
Having vehicles		
Do not have	72	83.7
Motorcycle	9	10.5
Car/truck	4	4.7
Motorcycle and car	1	1.2
Having radio	35	40.7
Having TV	30	34.9
History of IRS (in the last 12 months)		
No	59	68.6
Yes	21	24.4
Do not remember	6	7.0
Mosquito bed nets		
Having bed nets	45	52.3
Using bed nets	28	32.6
Source of bed nets (governmental)	45	100.0
What do you do first when have a fever?		
Go to clinic/hospital	47	54.7
Self-treatment	18	20.9
Do nothing	21	24.4

*IRS* indoor residual spraying

^a^Mean (SD)

^b^Median (interquartile range)

Overall, asexual parasite clearance was rapid as no patient was found parasitaemic on days 3 and 7. By day 1, 51 patients (59.3 %) were cleared of parasites and only four (4.7 %) patients still had microscopically detectable parasitaemia on day 2 which subsequently cleared on day 3. Asexual parasites with fever reappeared (recrudescent/re-infection cases) on day 14 in two patients, on day 21 in another, and finally on day 28 in two further patients (Additional file 2). Interestingly, these five patients were from Khamis Bani-Saad District of Al-Mahweet province, from the following villages: two from Deer Shareef, one from Al-Meshaahra, one from Shat Hajal, and one from Al-Rufae. Table 2 shows the parasitological and clinical outcomes reported among the patients treated with AS + SP.

**Table 2:** Summary of parasitological and clinical outcomes among patients treated with AS + SP after 28 days of follow-up

Classification	Without PCR correction		PCR-corrected	
	Number	Proportion (95 % CI)	Number	Proportion (95 % CI)
ETF	0	0 (0)	0	0 (0)
LCF	5	5.8 (1.9, 13.0)	3	3.5 (0.7, 9.9)
LPF	0	0 (0)	0	0 (0)
ACPR	81	94.2 (87.0, 98.1)	83	96.5 (90.1, 99.3)
Total analysis	86			
WTH	1			
LFU	2	3.4		
Total	89			

*CI* confidence interval, *ACPR* adequate clinical and parasitological response, *ETF* early treatment failure, *LCF* late clinical failure, *LPF* late parasitological failure, *LFU* loss to follow-up, *WTH* withdrawn

**Fig. 2:**
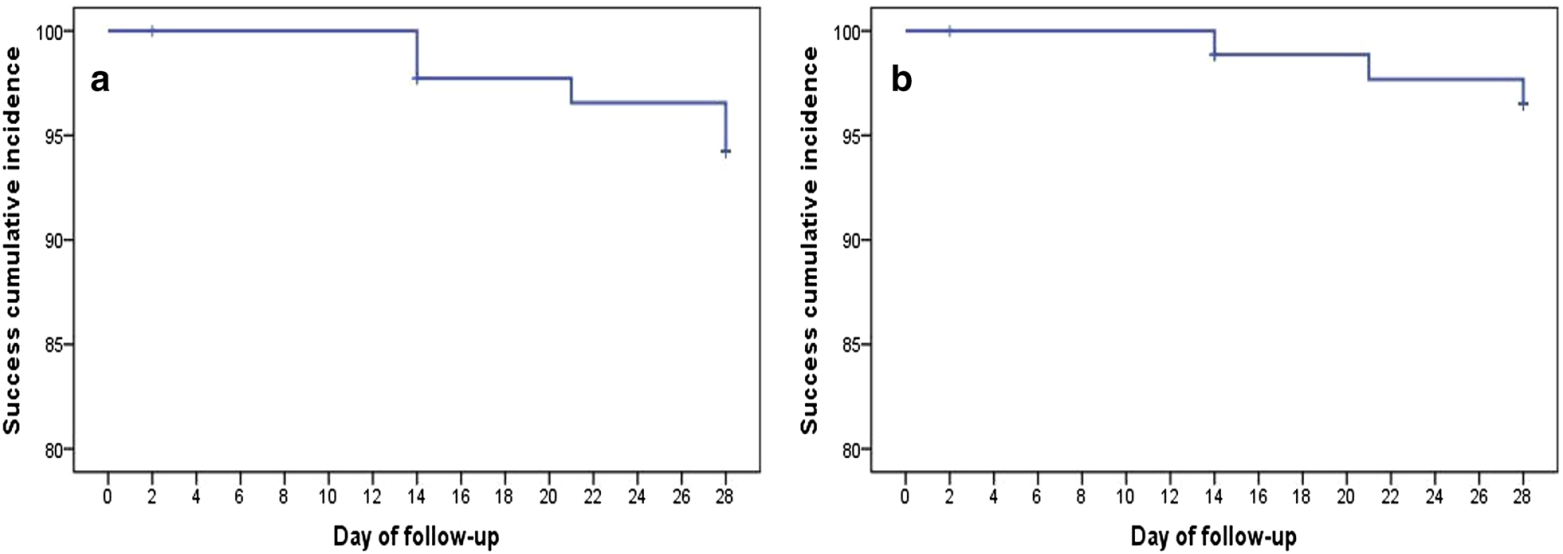
Kaplan-Meier curves showing treatment success cumulative proportion for the population under study for AS + SP up to day 28 of follow-up. (**a**) PCR-uncorrected, and (**b**) PCR-corrected

After Kaplan–Meier analysis, therapeutic outcome of AS + SP was 94.2 % (before PCR-correction) with 81 cases showing adequate clinical and parasitological response (Fig. 2a). After PCR-correction, three cases were classified as late clinical failure (recrudescences) as the genotypes of the parasites from the pre-treatment samples (i.e. samples collected on day 0 before the first dose of treatment was administered) and those on the day of parasite reappearance were similar. The remaining two cases were classified as new infections (Additional file 3). Thus, the PCR-corrected cure rate increased to 96.5 % (Fig. 2b). All reported treatment failures were observed in the age groups under the age of 15 (four below 5 years of age and one aged between five to fifteen). Overall, the mean age of the five patients who were classified as LCF was significantly lower than patients who were classified as ACPR (3.3 vs. 12.9 years; *P* = 0.006). Moreover, almost similar mean baseline parasitaemia was reported between the LCF and the ACPR cases (8375 vs. 8188 parasites/µL; *P* = 0.612).

### Molecular markers of drug resistance

*Pfk13*-*propeller* sequences were obtained from all 86 (100 %) isolates collected in this study. None of the study isolates analysed for mutations of the *pfK13* propeller domain (including the three recrudescent isolates) carried any mutations at the 39 codons previously published to be associated with artemisinin resistance [6, 10, 39].

*Pfdhfr* and *pfdhps.* For *pfdhfr*, sequences were obtained from 81 (94.2 %) of the study isolates. Double mutations at S108N and N51I were observed in 53 isolates (65.4 %), and all the other codons (16, 50, 59, and 164) were found to be wild type. Thus, the ACICNI *pfdhfr* haplotype was predominant (65.4 %) over the wild-type ACNCSI haplotype. For *pfdhps*, sequences were obtained from 76 isolates (88.4 %), and no mutations were observed for codons 436, 437, 540, 581, and 613, though mutations were in two other codons: 640 (n = 6) and 645 (n = 4). The distribution of the *pfdhfr* double-mutated haplotype frequency was found significantly associated with the districts and sites from which the participants were recruited: in 87.5 and 80.4 % of those from Ad-Dahee and Khamis Bani-Saad, respectively, while patients from Bajil districts harboured parasites that were mostly wild type (5.9 % with mutated haplotypes). Parasites obtained from patients from the villages of Al-Humarah (AdDahee) and Shat Hajal (Khamis Bani-Saad) all carried the double mutation. No association was reported between the frequency of *pfdhfr* mutations and other variables such as sex and age, admission parasite density, or gametocytaemia.

### Gametocytaemia

**Fig. 3:**
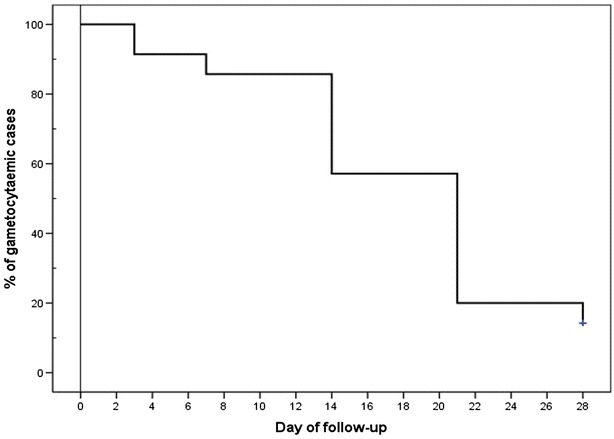
Kaplan-Meier curves showing time to disappearance of microscopic gametocytaemia in gametocytaemic individuals at enrolment and following AS + SP treatment. (n = 35)

Gametocytes were observed in 69 of the 189 persons found infected with *P. falciparum* during the initial survey, and a similar proportion of the patients recruited (35/86) were also gametocytaemic at the initiation of AS + SP treatment. Most of these patients still harboured gametocytes by day 7 post-treatment, and 14 % were still gametocytaemic at the end of the trail follow-up period (Fig. 3).

## Discussion

ACT is now the recommended first-line treatment in nearly all the malaria-endemic countries of the world (all Caribbean and Central American countries, except Panama, still rely on chloroquine + primaquine). High levels of resistance to SP have generally precluded the selection of SP as the ACT companion drug, and the AS + SP combination has been uniquely adopted in countries of WHO’s Eastern Mediterranean Region (except for Djibouti) and India where it is supplemented by primaquine, but it is administered alone in Azerbaijan, Somalia, Sudan, and Yemen. Increased frequencies of mutations associated with SP failure have been recorded in Sudan [40], and in particular in Somalia where unacceptable levels of treatment failure (22 %) were recorded in one area [41].

The study presented here indicates that the efficacy of AS + SP treatment is still high in Yemen (>95 %), which concords with the only other in vivo efficacy trial that was recently conducted in the same country [26]. In both studies, asexual parasitaemia was cleared by day 3 post-treatment, and only a few PCR-confirmed cases of true recrudescence were observed. In neither study were drug levels in those where treatment failed measured, and drug absorption or metabolism as a basis for failure could not be formally excluded. It is noteworthy that the three true recrudescences detected in the current study originated from the district with the highest percentage of malaria infection in surveyed residents (Khamis Bani-Saad district in Al-Mahwit province) and where all the isolates had the *pfdhfr* double mutation. Given high cure rate and the absence of resistance-associated mutations in the *pfdhps* gene and the rarity of triple mutations in the *pfdhfr* gene in Yemen [30], a recommendation to abandon SP as the partner drug is not warranted in Yemen. Nonetheless, constant monitoring of AS + SP efficacy should be a priority, not least because SP tablets are available in pharmacies as well as ordinary stores in rural areas, where they are kept at home and used for self-medication, a practice that could contribute to a rapid emergence of SP resistance. Previous studies revealed that CQ, quinine and SP are the most available anti-malaria drugs in private drug stores in Yemen. They are prescribed for uncomplicated falciparum malaria, and when it is financially possible they are used for self-administration, particularly in rural areas [42, 43].

In neighbouring and other Eastern Mediterranean countries, a similar profile of *pfdhfr* and *pfdhps* mutations has been reported in the Jazan region in southwest Saudi Arabia that shares borders with this study area [44], and in Afghanistan where the *pfdhfr* double mutation (C59R, S108N) predominates [45]. Despite the high frequency of mutations in both *pfdhfr* and *pfdhps* genes reported in Pakistan, the triple *pfdhfr* mutant was not observed, though 52 % (88/170) of the isolates studied harboured the *pfdhps* A437G mutation with the double mutated (C59R + S108N) *pfdhfr* [46]. Studies from south-eastern Iran showed an initial steady reduction in the prevalence of *pfdhfr*  +  *pfdhps* triple mutations (*pfdhfr* C59R + S108N and *pfdhps* A437G) during 2008–2010 following the adoption of AS + SP in 2007 [47], but a significant increase in their prevalence was later reported in the same area during 2012–2014 [48]. Ultimately, SP remains a suitable partner drug to AS for the treatment of uncomplicated falciparum malaria, and regular monitoring for the increase in resistance-associated mutations is warranted.

This first investigation of the *pfk13* propeller domain in *P. falciparum* from Yemen indicated that the parasites are still free of the mutations associated with artemisinin resistance in Asia. This might be due to the relatively short exposure of the parasites to artemisinin as well as the use of SP as the partner drug. Indeed, although ACT was introduced in 2009, its distribution at district level was gradual and its widespread use is slowed by continuing availability and prescription of chloroquine in both urban and rural areas [30, 42, 43]. Nonetheless, the potential for rapid selection and spread of mutants should not be underestimated and *pfk13* should be regularly monitored in Yemen.

In the context of sustainable malaria control, the use of SP for treatment suffers from a major disadvantage, namely a poor efficacy in eliminating the sexual stages. Thus, when SP was assessed in in vivo trials alone or in combination with artemisinins, the rapid clearance of asexual parasites was not observed for the gametocytes that often persisted for many days, sometimes throughout the follow-up period, in the circulation and remained infectious to mosquitoes. It is likely that the prevalence of post-treatment gametocytaemia was underestimated when microscopy alone was used to detect the gametocytes. Indeed, numerous studies showed that molecularly detectable sub-microscopic gametocytes are common following treatment and could be sufficient to sustain transmission [49–54]. In Yemen this is exacerbated by a delay in seeking treatment [55], probably due to limited financial resources. In the current study, approximately half of the persons surveyed admitted that they self-medicated or did not seek treatment when fever developed (Table 1), and this is reflected in the high proportion of screened patients (69/189, 36.5 %) that were gametocytaemic at the time of admission. A pattern similar to the one observed in this study was obtained for the sexual stages in the other in vivo trial of AS + SP efficacy conducted in Yemen [26]. Given that artemisinin has limited activity against mature gametocytes [56, 57], the use of AS + SP in Yemen might provide a selective advantage for the transmission of SP-resistant parasites [58, 59].

The data presented here reinforce the suggestion that the fate of the sexual stages should be considered as an additional factor to measure in standardized protocols of in vivo anti-malarial efficacy studies when the objectives of the malaria control programme also aim to reduce transmission [31]. Furthermore, it will be now judicious to recommend the addition of primaquine to the standard AS + SP first-line treatment in Somalia, Sudan and Yemen. Concerns over primaquine dose-dependent haemolytic effects in persons with glucose-6-phosphate dehydrogenase (G6PD) [60] are allayed by recent WHO guidelines that indicate that a single primaquine dose (0.25 mg base/kg) concomitantly administered on the first day of ACT treatment, is both effective in blocking transmission and unlikely to cause serious toxicity in individuals with any of the G6PD-deficiency variants, obviating the need for systematic and onerous G6PD testing [59, 61, 62].

## Conclusions

The data from Yemen indicates that AS + SP is still efficacious for treatment of uncomplicated falciparum malaria. Double mutant *pfdhfr* genotype was found in 65.4 % of the isolates while no mutation was reported in the *pfdhps* as well as *pfk13* genes. Moreover, persistent gametocytaemia after AS + SP treatment suggests the addition of primaquine in order to reduce malaria transmission sustainably. This is all the more important in Yemen, where disruption of the health infrastructure and population displacements due to the current political situation are likely to lead to an exacerbation of malaria and to threaten the surrounding countries in the Arabian Peninsula.

## Additional files

10.1186/s12936-016-1344-0 Oligonucleotide sequences and cycling conditions for genotyping *msp1*, *msp2*, *glurp,* *pfk13*, *pfdhfr* and *pfdhps*.
10.1186/s12936-016-1344-0 Parasitaemia clearance and re-appearance of the five recrudescent/re-infection cases.
10.1186/s12936-016-1344-0 Genotyping data for the five patients with renewed clinical activity during follow-up.
