# Supplementary material for: Sustained efficacy of artesunate-sulfadoxine-pyrimethamine against Plasmodium falciparum in Yemen and a renewed call for an adjunct single dose primaquine to clear gametocytes
Source: Malar J. 2016 May 27;15:295. doi: 10.1186/s12936-016-1344-0 (PMC4882835; doi:10.1186/s12936-016-1344-0)
Supplement: Supplementary file 1 — 10.1186/s12936-016-1344-0 Oligonucleotide sequences and cycling conditions for genotyping msp1, msp2, glurp, pfk13, pfdhfr and pfdhps. [file 12936_2016_1344_MOESM1_ESM.pdf]

Additional file 1

Oligonucleotide sequences and cycling conditions for genotyping *msp1*, *msp2* and *glurp*

	Gene/Family	Primer name	Primer sequence	Thermal conditions
Primary reaction	MSP-1	M1-OF	5’ -CTAGAAGCTTTAGAAGATGCAGTATTG-3’	1 cycle (95°C/5 min, 58°C/2 min, 72°C/2 min)<br>25 cycles (94°C/1 min, 58°C/2 min, 72°C/2 min)<br>Extension at 72°C/2 min<br>Annealing at 58°C/2 min<br>Final extension at 72°C/5 min
		M1-OR	5’ -CTTAAATAGTATTCTAATTCAAGTGGATCA-3’	
	MSP-2	M2-OF	5’ -ATGAAGGTAATTAAAACATTGTCTATTATA-3’	
		M2-OR	5’ -CTTTGTTACCATCGGTACATTCTT-3’	
	GLURP	G-OF	5’ -TGAATTTGAAGATGTTCACACTGAAC-3’	
		G-OR	5’ -GTGGAATTGCTTTTTCTTCAACACTAA-3’	
Secondary reaction	MSP1-K1	M1-KF_FAM	/56-FAM/AAATGAAGAAGAAATTACTACAAAAGGTGC	
		M1-KR	5’ -GCTTGCATCAGCTGGAGGGCTTGCACCAGA-3’	1 cycle (95°C/5 min, 61°C/2 min, 72°C/2 min)<br>25 cycles (94°C/1 min, 61°C/2 min, 72°C/2 min)<br>Extension at 72°C/2 min<br>Annealing at 61°C/2 min<br>Final extension at 72°C/5 min
	MSP1-MAD20	M1-MF_FAM	/56-FAM/AAATGAAGGAACAAGTGGAACAGCTGTTAC	
		M1-MR	5’ -ATCTGAAGGATTTGTACGTCTTGAATTACC-3’	
	MSP1-RO33	M1-RF_FAM	/56-FAM/TAAAGGATGGAGCAAATACTCAAGTTGTTG	
		M1-RR	5’ -CATCTGAAGGATTTGCAGCACCTGGAGATC-3’	
	MSP2-FC27	M2-FCF_FAM	/56-FAM/AATACTAAGAGTGTAGGTGCARATGCTCCA	
		M2-FCR	5’ -TTTTATTTGGTGCATTGCCAGAACTTGAAC-3’	
	MSP2-IC	M2-ICF_FAM	/56-FAM/AGAAGTATGGCAGAAAGTAAkCCTYCTACT	
		M2-ICR	5’ -GATTGTAATTCGGGGGATTCAGTTTGTTCG-3’	
	GLURP	G-NF	5’ -TGTTCACACTGAACAATTAGATTTAGATCA-3’	
		G-OR	5'-GTGGAATTGCTTTTTCTTCAACACTAA-3’	

Oligonucleotide sequences and cycling conditions for *PfK13, Pfdhfr* and *Pfdhps*

Gene	PCR	Primer	Sequence	Size	Thermal conditions
Pf-Kelch 13	Single run	K13-F<br>K13-R	GTTGGTGGAGCTATTTTTGAAACATCTAG<br>GCCAAGCTGCCATTCATTTGTATC	1062	94°C /5 min<br>40 cycles (94°C/30 sec, 60°C/90 sec, 72°C/90 sec)<br>72°C/10 min
Pfdhfr	Primary	Amp1<br>Amp2	TTTATATTTTCTCCTTTTTA<br>CATTTTATTATTCGTTTTCT	718	94°C/5 min<br>30 cycles (94°C/30 sec, 49°C/60 sec, 72°C/60 sec)<br>72°C/5 min
	Secondary	SP1<br>SP2	ATGATGGAACAAGTCTGCGAC<br>ACATTTTATTATTCGTTTTC	700	94°C /5 min<br>25 cycles (94°C/30 sec, 49°C/60 sec, 72°C/60 sec)<br>72°C/5 min
Pfdhps	Primary	PS1-F<br>O2	GAATTTTTATCCATTCCTCATG<br>TTCCTCATGTAATTCATCTGA	1028	94°C /5 min<br>30 cycles (94°C/60 sec, 56°C/2 min, 72°C/60 sec)<br>72°C/5 min
	Secondary	PSA-F<br>O2	GTATACAACACACAGATATAG<br>TTCCTCATGTAATTCATCTGA	1005	94°C/5 min<br>25 cycles (94°C/60 sec, 56°C/2 min, 72°C/60 sec)<br>72°C/5 min
