# Supplementary material for: Sustained efficacy of artesunate-sulfadoxine-pyrimethamine against Plasmodium falciparum in Yemen and a renewed call for an adjunct single dose primaquine to clear gametocytes
Source: Malar J. 2016 May 27;15:295. doi: 10.1186/s12936-016-1344-0 (PMC4882835; doi:10.1186/s12936-016-1344-0)
Supplement: Supplementary file 2 — 10.1186/s12936-016-1344-0 Parasitaemia clearance and re-appearance of the five recrudescent/re-infection cases. [file 12936_2016_1344_MOESM2_ESM.pdf]

Additional file 2 Parasitaemia clearance and re-appearance of the five recrudescent/re-infection cases

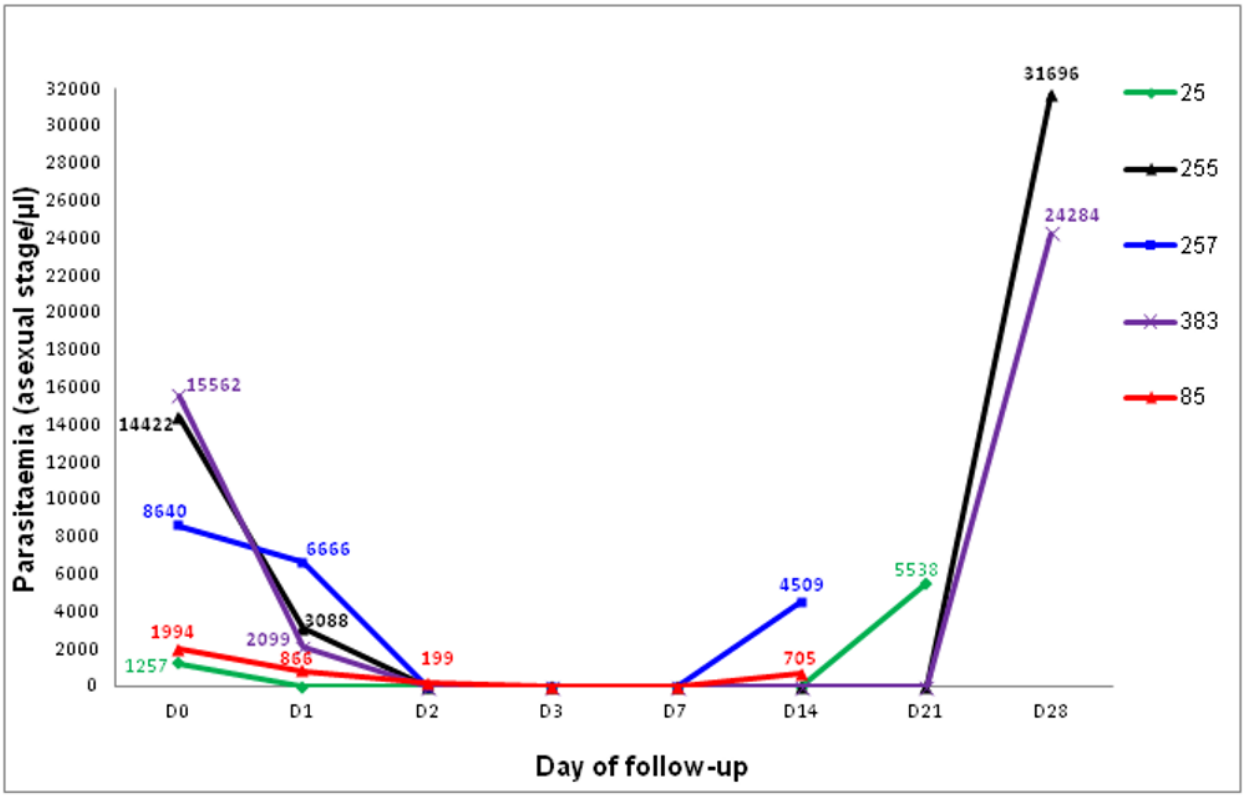

Patient identification numbers are in the panel on the right.
