# Supplementary material for: Sustained efficacy of artesunate-sulfadoxine-pyrimethamine against Plasmodium falciparum in Yemen and a renewed call for an adjunct single dose primaquine to clear gametocytes
Source: Malar J. 2016 May 27;15:295. doi: 10.1186/s12936-016-1344-0 (PMC4882835; doi:10.1186/s12936-016-1344-0)
Supplement: Supplementary file 3 — 10.1186/s12936-016-1344-0 Genotyping data for the five patients with renewed clinical activity during follow-up. [file 12936_2016_1344_MOESM3_ESM.pdf]

Additional file 3 Genotyping data for the five patients with renewed clinical activity during follow-up*

ID	Day	*msp1*			*msp2*		*glurp*	Outcome
		K1	MAD20	RO33	FC27	IC		
25	0	180	-	-	300	-	1,000	Recrudescence
	21	180	-	-	300	-	1,000	
85	0	-	-	150	450	-	900	New infection
	14	200	-	-	-	500	1,000	
255	0	180	-	-	-	500	1,000	Recrudescence
	28	180	-	-	-	500	1,000	
257	0	180	-	150	300	600	900	Recrudescence
	14	180	-	150	300	600	900	
383	0	200	-	-	350	-	900	New infection
	28	-	230	-	280	-	900	

* The frequency of the recrudescent allelic variants observed in the study area was as the following: MSP1-K1-180-200 bp=12.5%; MSP1-MAD20-230 bp=32.5%; MSP1-RO33 150 bp=25%; MSP2-FC27-280-300 bp=13.2%; MSP2-FC27-350 bp=27.9%; MSP2-FC27-450 bp=13.2%; MSP2-IC-500 bp=5.9%; MSP2-IC-600 bp=39.7%; GLURP 900 bp=33%; GLURP 1,000 bp=30.5%.
